# Albumin-based nanoparticles encapsulating SN-38 demonstrate superior antitumor efficacy compared to irinotecan

**DOI:** 10.1080/10717544.2025.2545519

**Published:** 2025-08-17

**Authors:** Guojun Xiong, Shengxi Li, Andreas G. Schätzlein, Ijeoma F. Uchegbu

**Affiliations:** a School of Pharmacy, University College London, London, UK; b Nanomerics Ltd., London, UK; c Wolfson College, University of Cambridge, Cambridge, UK

**Keywords:** Protein drug delivery system, lactone–carboxylate equilibrium, SN-38 loaded nanoparticles, HSA–PLA, Human serum albumin

## Abstract

Efficient formulation of SN-38 for broad-spectrum chemotherapy remains an unmet medical need. The limited solubility of SN-38 in both aqueous and organic solvents poses a major challenge for formulation development. As a result, the predominant strategy, polymer-SN-38 drug conjugates, often involves complex synthetic procedures and low drug loading (1–5% w/w). Such limitations hinder their large-scale production and clinical translation. In this study, we developed an encapsulation strategy that utilizes the reversible lactone–carboxylate equilibrium of SN-38 to simplify the formulation process and achieve enhanced drug loading. The major issue of SN-38 solubility in organic solvents was effectively addressed by sodium hydroxide (NaOH)-induced conversion of the lactone to the carboxylate form. We have demonstrated that SN-38 carboxylate, once encapsulated within human serum albumin–polylactic acid (HSA–PLA) nanoparticles, retains its reversibility and can be converted back to the active lactone form simply by the addition of hydrochloric acid (HCl). The drug loading capacity of SN-38 in the HSA–PLA nanoparticles was increased to 19% w/w. *In vitro* cytotoxicity assays confirmed that HSA–PLA (SN-38) nanoparticles exhibited significantly lower IC_50_ values (0.5–194 nM) across multiple cancer cell lines compared to the clinical standard, irinotecan (CPT-11), indicating superior potency under physiological conditions. *In vivo* studies in 4T1 and MDA-MB-231 tumor-bearing mice further validated the enhanced therapeutic efficacy of this formulation. Overall, this study presents a promising alternative strategy for SN-38 delivery via encapsulation rather than polymer–drug conjugation, significantly simplifying the formulation process and enhancing the translational potential of SN-38 for broad chemotherapeutic applications.

## Graphical Abstract

**Figure:**
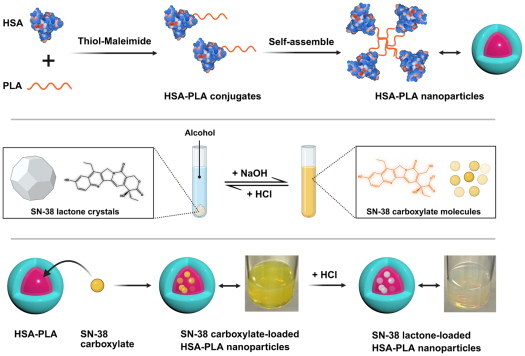

## 1. Introduction

**Figure 1.**
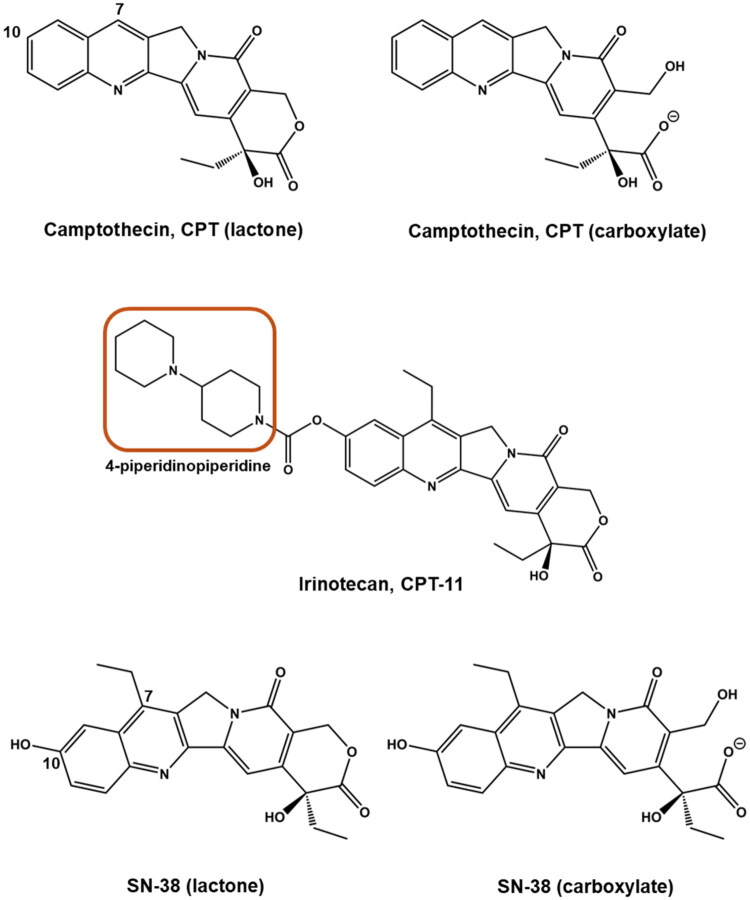
Chemical structures of camptothecin (CPT) and its derivatives. The structural forms of camptothecin (CPT) and its derivatives, including irinotecan (CPT-11) and SN-38. Camptothecin exists in two forms: lactone (active) and carboxylate (inactive). Similarly, SN-38, the active metabolite of irinotecan, also exhibits a lactone–carboxylate equilibrium. The structure of irinotecan (CPT-11) includes a 4-piperidinopiperidine moiety (highlighted), which forms salts with hydrochloride to enhance water solubility. The 7-ethyl and 10-hydroxy groups on SN-38 inhibit the transformation to its carboxylate form under physiological conditions when compared with CPT.

In the last century, camptothecin (CPT) was isolated from the stems of the *Camptotheca acuminata* and was found to be effective against cancer by inhibiting topoisomerase I (Top I) activity (Wall et al. 1966). Initial clinical trials of CPT revealed that the lipophilic CPT (lactone) might be more effective against cancer than its ionized form (carboxylate) (Zhang et al. 2013). The chemical structure of these two epimers is shown in Figure 1. However, as formulating the lipophilic lactone was not overly successful, substantial efforts were made to develop CPT analogues that enhance the solubility of its lactone form (Li et al. 2017). This gave rise to irinotecan (CPT-11), a CPT derivative, which was formulated as a hydrochloride (HCl) salt and approved for clinical use by the United States Food and Drug Administration (US FDA) in 1996 (Venditto and Simanek 2010). The addition of the 4-piperidinopiperidine group in CPT-11 (highlighted in Figure 1) provides sites for salt formation with HCl, which addressed the solubility issues. However, the efficacy of irinotecan is compromised because the active ingredient, SN-38 (7-ethyl-10-hydroxycamptothecin) needs to be released from CPT-11 through hydrolysis by carboxylesterase enzymes (Innocenti et al. 2009). The anticancer potency of SN-38 lactone is, in fact, 100–1000 times higher than that of CPT-11 *in vitro* (Qi et al. 2024).

Nanoscale drug delivery systems provide an alternative approach for formulating hydrophobic drugs without additional toxicity or compromising efficacy, especially protein-based delivery systems (Zhang et al. 2023; Jiang et al. 2024; X. Man et al. 2024; X.-Y. Man et al. 2024), such as Abraxane and Trodelvy. Many efforts have been made to formulate CPT and SN-38 into more effective and stable medicines (Yan et al. 2017), various formulations of SN-38 have been evaluated in preclinical and clinical studies (J. Yang et al. 2023; Qi et al. 2024). However, the extremely low solubility of CPTs (in their lactone forms) in water as well as in clinically available solvents and oils complicates their formulation methods (J. Yang et al. 2023). The majority of SN-38 formulations are based on covalently conjugating SN-38 to polymers, proteins, lipids, or antibodies, which make SN-38 a prodrug once again and present with a drawback of low drug loading. For example, Sepehri et al. designed a human serum albumin–SN-38 conjugate (HSA–SN-38) with a drug content of 4.4% w/w (Sepehri et al. 2014).

In this work, we did not exploit the drug delivery strategy of conjugating SN-38 to polymers, not only because of the low drug loading, but also the complexity of the chemical modification procedures, which usually requires more than four steps of chemical synthesis (J. Yang et al. 2023; Qi et al. 2024). This complex process inevitably results in losses at each step, leading to final yields of less than 50% (Zhang et al. 2013). Additionally, the conjugation process requires the use of toxic solvents and catalysts, such as hexane and 4-(dimethylamino)pyridine (DMAP), as well as multiple purification steps. These factors may pose challenges in terms of reproducibility, hinder scale-up production, and potentially complicate toxicological outcomes in later stages. For example, Lu et al. chemically modified SN-38 through more than four synthetic steps to obtain SN-38 prodrugs (overall yield < 50%) capable of both covalent and non-covalent binding to HSA, with a final drug loading of less than 2% (w/w) (Lu et al. 2025). Notably, their non-covalently bound SN-38-HSA formulation exhibited superior anticancer efficacy compared to its covalently bound counterpart. Their findings indicated that the covalent conjugation of SN-38 to a polymer provided no improvement in efficacy and further suggested that SN-38–polymer conjugates may not represent an optimal formulation strategy (Lu et al. 2025).

Therefore, encapsulating SN-38 or enabling its non-covalent binding within HSA nanoparticles may represent a more efficient and clinically translatable strategy than covalent conjugation. However, the low drug loading (1% w/w and 8% w/w) remains a challenge, as reported in other studies, involving the encapsulation of SN-38 in albumin nanoparticles (Lin et al. 2019; S.-J. Yang et al. 2023).

The poor solubility of SN-38 lactone in various solvents is a major factor contributing to the low drug loading in encapsulation methods. In practice, we found that the solubility of SN-38 carboxylate in alcohols was significantly increased, reaching up to 20 mg/mL in methanol (MeOH). The dissolved SN-38 carboxylate in MeOH was reversibly converted to SN-38 lactone upon the addition of hydrochloric acid (HCl), resulting in its precipitation within the methanolic solution. This finding corresponds with others’ work showing that SN-38 solubility can be increased in alkaline solutions (Zhang et al. 2004). Therefore, we proposed an approach for effectively loading SN-38 into HSA nanoparticles by temporarily utilizing the high solubility of SN-38 carboxylate in an alcoholic solution and then converting it to SN-38 lactone after encapsulation.

In this study, human serum albumin–polylactic acid (HSA–PLA) nanoparticles were employed to validate our SN-38 drug loading strategy. HSA–PLA is an amphiphilic and self-assembling nanoplatform specifically designed for the universal delivery of hydrophobic drugs (Xiong, Li, et al. 2025). Compared to plain HSA nanoparticles, we performed site-specific conjugation of a hydrophobic polymer (PLA) to the Cys34 residue of HSA via a thiol-maleimide reaction. The resulting construct forms a distinct hydrophobic core that enables the universal encapsulation of hydrophobic drugs. Detailed characterization of this system has been reported in our previous work (Xiong, Li, et al. 2025). Therefore, SN-38 carboxylate was efficiently encapsulated into the hydrophobic cores of the HSA–PLA nanoparticles and subsequently converted back to SN-38 lactone within the nanoparticles upon the addition of HCl. Compared to other SN-38 conjugation strategies, our approach of encapsulating SN-38 into HSA–PLA nanoparticles offers several advantages, including time- and cost-efficiency, a simplified preparation process, ease of scale-up, high reproducibility, and a notably high drug loading of 19% (w/w). The use of the SN-38 lactone–carboxylate equilibrium technique significantly enhances the translational potential of SN-38 drug delivery systems for cancer therapy.

## 2. Methods and materials

### 2.1. Preparation of SN-38 loaded HSA–PLA NPs and CPT-11 formulation

HSA–PLA nanoparticles were prepared as previously described (Xiong, Li, et al. 2025).

SN-38 (**#**459090010, Thermo Fisher Scientific, Loughborough, UK, 20 mg) was dissolved in MeOH containing sodium hydroxide (**#**32213-2.5L-M, Sigma Aldrich, Gillingham, UK, 1 mL, 0.22% w/v sodium hydroxide (NaOH)). The resulting methanolic SN-38 solution was added to a HSA–PLA water dispersion (0.8% w/v, 10 mL). SN-38 was encapsulated into HSA–PLA nanoparticles using a probe sonicator (Soniprep 150 Plus, MSE, London, UK) in an ice bath. The sonication was performed at level 5 amplitude for two 5-minute sessions, with a 3-minute rest in between. Subsequently, HCl (0.1 M, 0.55 mL) was added to the HSA–PLA (SN-38) nanosuspension to adjust the pH value to 7.0. Trehalose (**#**90210, Sigma Aldrich, Gillingham, UK, 100 mg) was dissolved in the above HSA–PLA (SN-38) dispersion as a cryoprotectant. After filtration with a 0.45 µm filter (**#**SLHA033SS, Merck, Gillingham, UK), the unencapsulated SN-38 was removed. Subsequently, the purified HSA–PLA (SN-38) nanosuspension was frozen overnight at −20 °C, followed by a two-day lyophilization (ALPHA 1-4 LDplus, Martin Christ, Osterode am Harz, Germany). The lyophilized powder of HSA–PLA (SN-38) NPs was stored at a temperature of 4 °C until required for further studies.

CPT-11 standard formulation was prepared according to the indication of CAMPTOSAR (Lee et al. 2023). Briefly, CPT-11 (**#**B2293, ApexBio, Houston, TX, 20 mg) was dissolved in 1 mL of distilled water containing d-sorbitol (**#**BP439, Thermo Fisher Scientific, Loughborough, UK, 45 mg) and lactic acid (**#**252476, Sigma Aldrich, Gillingham, UK, 0.9 mg) in a water bath at 80 °C. After cooling down to room temperature, the pH value of the CPT-11 solution was adjusted to 3.0.

### 2.2. Determination of drug loading via HPLC

The lyophilized powder (2 mg) was dispersed in MeOH aqueous solution (60% v/v, 5 mL) containing NaOH (0.1% w/v). The resulting mixture was subjected to two minutes of vortex mixing and bath sonication. Afterwards, this mixture was filtered using a 0.2 μm PTFE filter.

The drug concentrations in the filtrates were determined using a high-performance liquid chromatography (HPLC) system (Agilent Technologies 1200 series, Santa Clara, CA) equipped with a reversed-phase (RP) column (#993967-902, ZORBAX Eclipse XDB-C18, 4.6 × 150 mm, 5 µm). The mobile phase consisted of 40% water (0.1% w/v NaOH) and 60% MeOH (0.1% w/v NaOH). HPLC parameters were listed as follows.

**Table:** 

Parameters	Values
Flow rate (mL/min)	0.5
Run time (min)	5
Detection wavelength (nm)	229
Injection volume (μL)	10
Column temperature (°C)	25
Calibration curve equation	*y* = 108,805*x* + 66.91 (where *x* = concentration of SN-38 mg/mL)
Correlation coefficient	0.9998

The 2 mg lyophilized powder contains SN-38, HSA–PLA NPs, trehalose, and NaCl (derived from NaOH + HCl).

Here, we define:

- *a* = quantity of SN-38 in 2 mg lyophilized powder, measured by HPLC;
- *b* = quantity of trehalose in 2 mg lyophilized powder;
- 0.8*b* = quantity of HSA–PLA NPs in 2 mg lyophilized powder;
- 0.03 mg ≈ quantity of NaCl in 2 mg lyophilized powder.

Therefore, *b* can also be determined as (2 − 0.03 − *a*)/1.8.

The drug loading capacity (LC%) and encapsulation efficiency (EE%) can be estimated using the following equations:

(1) LC%=aa+0.8b×100

(2) EE%=LC%20%×100

### 2.3. Dynamic light scattering (DLS)

The hydrodynamic diameter, zeta potential, and polydispersity index (PDI) of the HSA–PLA (SN-38) NPs were determined using a Malvern Nano-ZS instrument (Malvern Panalytical Ltd., Malvern, UK). Lyophilized powder (4 mg) was dispersed in 2 mL of distilled water, pH was balanced between 7.0 and 7.2. Three different batches of HSA–PLA (SN-38) NPs were measured in triplicate, respectively.

### 2.4. Transmission electron microscopy (TEM)

A drop of HSA–PLA (SN-38) nanosuspensions (5 mg/mL) was placed onto a TEM grid. After five minutes, any excess liquid on the TEM grids was removed with tissue paper. Next, a droplet of neutral phosphotungstic acid solution (#79690, Sigma Aldrich, Gillingham, UK, 1% w/v) was added to each TEM grid. After one minute of staining, the grids were washed with distilled water. The prepared TEM samples were stored in a dark location until imaging could be performed.

### 2.5. X-ray powder diffraction (XRD)

The XRD method was adapted from previous work (Xiong, Li, et al. 2025). Briefly, HSA–PLA blank nanoparticle powder, SN-38-loaded HSA–PLA nanoparticle powder, SN-38 standard powder, and SN-38 + HSA–PLA NPs powder mixtures were meticulously ground and positioned onto the respective sample holders. Samples were scanned by an X-ray diffractometer (MiniFlex 600, Rigaku, Neu-Isenburg, Germany). The specific parameters: angle 2*θ* scanned from 3° to 60°, step size = 0.02°, rate of scan = 10°/min, X-rays (*λ* = 1.5418 Å) generated by a CuKα tube at 40 kV and 15 mA.

### 2.6. Cell culture

A549, PC3, BT549, HT29, A431, MIA PaCa-2, MDA-MB-231, and 4T1 cell lines were obtained from group stock purchased from ATCC (Glasgow, UK). The A2780 cell line was bought from Sigma Aldrich (Gillingham, UK).

A549, PC3, BT549, HT29, A2780, A431, and 4T1 cells were each cultured in a tissue culture flask with a vented cap using a complete medium composed of Advanced RPMI 1640 Medium (**#**12633012, Thermo Fisher Scientific, Loughborough, UK) supplemented with heat-inactivated fetal bovine serum (FBS, #F9665, Sigma Aldrich, Gillingham, UK, 1% v/v), Glutamax (#35050-038, Thermo Fisher Scientific, Loughborough, UK, 1% v/v), and penicillin/streptomycin (#15140-122, Thermo Fisher Scientific, Loughborough, UK, 1% v/v).

MIA PaCa-2 cells were maintained in a complete medium consisting of DMEM (**#**41965039, Thermo Fisher Scientific, Loughborough, UK) supplemented with heat-inactivated FBS (10% v/v), Glutamax (1% v/v), penicillin/streptomycin (1% v/v), and sodium pyruvate (**#**BW13-115E, LONZA, Slough, UK, 1% v/v) in a tissue culture flask with a vented cap. All cells were maintained at 37 °C in a humidified incubator with 5% CO_2_.

MDA-MB-231 cells were maintained in a non-filter capped tissue culture flask filled with the Leibovitz’s L-15 Medium (#11415-049, Thermo Fisher Scientific, Loughborough, UK), supplemented with heat inactivated FBS (10% v/v) and penicillin/streptomycin (1% v/v), incubated at 37 °C in CO_2_ free environment at 95% humidity.

### 2.7. Endocytic pathway of HSA–PLA nanoparticles in 4T1

Approximately, 1 × 10^5^ 4T1 cells were seeded on glass coverslips (#10553322, Fisher Scientific Ltd., Loughborough, UK) placed in six-well plates containing complete cell culture medium. Upon reaching ∼80% confluence, the cells were incubated with FBS-free medium at 37 °C in a 5% CO_2_ atmosphere for four hours to allow equilibration. Subsequently, cells were treated with a mixture of HSA–PLA (FITC) nanoparticles and Dextran, Texas Red (70 kDa, #D1830, Thermo Fisher Scientific, Loughborough, UK), both at a final concentration of 0.5 mg/mL, for one hour at 37 °C under 5% CO_2_.

After incubation, the nuclei were stained with Hoechst 33342 (#R37605, Thermo Fisher Scientific, Loughborough, UK) for 30 minutes. Cells were then fixed with paraformaldehyde solution (#40-7401-05, Severn Biotech Ltd., Kidderminster, UK) for 15 minutes on ice and mounted using mounting medium (#ab104135, Abcam, Cambridge, UK). Confocal imaging was performed using a laser scanning confocal microscope (ZEISS LSM 710, ZEISS, Oberkochen, Germany).

This method was adapted from our previous study, in which the successful encapsulation of fluorescein isothiocyanate (FITC) into HSA–PLA nanoparticles was demonstrated (Xiong, Schätzlein, et al. 2025). Based on this established approach, it was applied in the present study.

### 2.8. Cell apoptosis assay

MDA-MB-231 cells were seeded in six-well plates at a density of 1 × 10^5^ cells per well and cultured in 3 mL of complete medium at 37 °C in a CO_2_-free incubator. Upon reaching approximately 60% confluence, cells were treated with CPT-11 (200 nM), HSA–PLA (SN-38) nanoparticles (200 nM), or the corresponding vehicle (HSA–PLA) for 48 hours. Untreated cells served as the negative control. Each treatment was performed in triplicate.

After treatment, each group of cells was processed separately. Suspended cells from the culture medium were first collected, and the remaining adherent cells were detached using TrypLE Express Enzyme (**#**12604021, Thermo Fisher Scientific, Loughborough, UK). Cells from the same treatment group were centrifuged and the supernatant was discarded. The resulting cell pellet was resuspended in 0.5 mL of Annexin V Binding Buffer (**#**422201, BioLegend, London, UK). For each treatment group, 200 µL of the cell suspension was transferred into a 1.5 mL eppendorf tube for apoptosis staining. Subsequently, FITC-Annexin V (**#**640906, BioLegend, London, UK, 5 µL) and propidium iodide (PI) (**#**421301, BioLegend, London, UK, 5 µL) were added to each cell sample and incubated for 15 minutes at room temperature in the dark. Finally, cold Dulbecco’s phosphate-buffered saline (DPBS, **#**D8662, Sigma Aldrich, Gillingham, UK, 1 mL) was added to each cell sample and immediately analyzed using a flow cytometer (Attune NxT Flow Cytometer, Thermo Fisher Scientific, Loughborough, UK).

### 2.9. *In vitro* cytotoxicity

A549, PC3, BT549, HT29, A2780, A431, and MIA PaCa-2 cells were respectively each pipetted into 96-well plates at a density of 1000 cells per well in 100 µL of their respective complete culture media. These cells were incubated at 37 °C in a humidified incubator with 5% CO_2_ until they reached 50–60% confluency. Samples of CPT-11 standard and HSA–PLA (SN-38) NPs were prepared and diluted with corresponding complete medium to achieve a series of pre-determined concentrations. Then, 100 µL of the diluted samples were added to the corresponding cell wells and incubated with the cells for 48 hours. After treatment, these cells were recovered for a 12–48 hour in fresh complete medium, depending on cell confluency. After recovery, these cells were washed twice with cold PBS (**#**10010031, Thermo Fisher Scientific, Loughborough, UK, 0.2 mL, pH 7.4), followed by the addition of fresh complete medium (100 µL per well) and WST-1 reagent (**#**11644807001, Sigma Aldrich, Gillingham, UK, 5 µL per well) for further incubation. The absorbance of the formazan dye, indicating peak and background levels, was measured at 440 nm and 650 nm, respectively, after 0.5–4 hours of incubation, using a SPECTROstar Omega plate reader (BMG LABTECH, Aylesbury, UK). Each experiment was conducted in triplicate, and data were analyzed using Prism software (Irvine, CA).

### 2.10. *In vivo* anticancer study

All animal experiments were conducted in accordance with the UK Animals (Scientific Procedures) Act 1986 and approved by the University College London Animal Welfare and Ethical Review Body (AWERB). The studies were carried out under the UK Home Office Project License (PP4970379) and Personal License (I1827609). The authors adhered to the ARRIVE guidelines (https://arriveguidelines.org/) with all animal studies.

*Justification for use of animals*: Animal models are essential for evaluating therapeutic efficacy in a biologically relevant setting. The use of NOD-SCID mice is justified by their immunodeficient status, which permits the engraftment and growth of human tumor xenografts without immune rejection, enabling the study of human-specific cancer biology and drug response. Immunocompetent BALB/c mice were used to evaluate the antitumor efficacy of the formulations within a fully functional immune system, providing critical insights into their translational feasibility.

A total of 25 female mice were used in this work and were handled by the author, Guojun Xiong. All steps met the requirements for personal license (No. I1827609) and project license (No. PP4970379) holders as issued by the UK home office. Fifteen immunocompetent female mice (BALB/c, 8–10 weeks old, 20 ± 2 g; *n* = 15) were housed at five animals per cage and maintained by staff at the UCL School of Pharmacy Biological Services Unit. Ten immunocompromised female mice (NOD-SCID, 10 weeks old, 22 ± 2 g; *n* = 10) were housed at five animals per cage in the SCID room and maintained by staff at the UCL Institute of Child Health Biological Services Unit.

*Animal housing and welfare*: All animals were housed in individually ventilated cages (IVCs) in a temperature-controlled (22 ± 3 °C), humidity-controlled (60 ± 5%) facility with a 12-hour light/dark cycle. Mice had *ad libitum* access to standard chow and filtered water. Environmental enrichment was provided, including nesting materials and shelters, to reduce stress. All efforts were made to minimize animal suffering, including careful monitoring and humane endpoints.

*Anesthesia*: No anesthesia was required during the study, as all procedures involved were noninvasive or minimally invasive (e.g. subcutaneous injection and intravenous injection), and did not cause pain or distress to the animals.

*Euthanasia and humane endpoints*: Mice were humanely euthanized using a rising concentration of CO_2_ followed by cervical dislocation, as approved under Schedule 1 of the UK Home Office regulations. Mice were monitored daily, and humane endpoints were defined as a tumor volume of 1200 mm^3^, body weight loss exceeding 15%, ulceration persisting for more than three days, or signs of systemic illness. To prevent tumor volumes from exceeding the 1200 mm^3^ humane endpoint, animals were euthanized when tumor size reached approximately 600–1000 mm^3^.

#### 2.10.1. 4T1 tumor model

Female BALB/c mice (8–10 weeks, 20 ± 2 g, *n* = 15, Charles River Laboratories, Margate, UK) were subcutaneously injected with 4T1 cells (2 × 10^6^ cells/100 μL of PBS) in the right flank. Seven to ten days after the injection of the 4T1 cells, tumor volume reached approximately 100 mm^3^.

These 4T1 bearing mice were randomly divided into three groups with five mice in each group (*n* = 5). The groups were as follows: control group treated with HSA–PLA blank nanoparticles, CPT-11 group (equivalent to CPT-11 40 mg/kg), and HSA–PLA (SN-38) group (equivalent to SN-38 40 mg/kg). Mice received two intravenous injections on day 0 and day 3. The 4T1 tumor-bearing mice were euthanized using a Schedule 1 method (a rising concentration of CO_2_ followed by cervical dislocation), in accordance with the Animals (Scientific Procedures) Act 1986.

#### 2.10.2. MDA-MB-231 tumor model

NOD-SCID female mice (10 weeks, 22 ± 2 g, *n* = 10, Charles River Laboratories, Margate, UK) were subcutaneously injected with MDA-MB-231 cells (1 × 10^7^/100 μL PBS) in the right flank. Once the tumor volumes reached approximately 100 mm^3^, the mice were randomly allocated into two groups (with five mice in each group, *n* = 5). These groups were designated as follows: CPT-11 group and HSA–PLA (SN-38) group. Mice were intravenously administered with 0.1 mL of the corresponding drugs at an equivalent dose of 20 mg/kg on day 0, 3, 6, 9, and 12. Data on a non-treated group from previous work (Xiong, Schätzlein, et al. 2025) were used as a comparator.

The tumor diameters were measured using a vernier caliper and mice body weights were recorded using a laboratory balance. Mice were euthanized with a Schedule 1 method if the tumor volume reached 600–1000 mm^3^, there was a body weight loss exceeding 15%, or tumor ulceration persisted for more than three days.

The tumor volume was calculated through the following equation:

(3) Tumor volume=0.5×length×width2

### 2.11. Statistics

Data are presented as means ± standard deviation (SD). A multiple *t*-test (with fewer assumptions) was used to analyze IC_50_ values. One-way ANOVA was employed to analyze differences in the proportion of apoptotic cells between groups. Two-way ANOVA was applied for the statistical analysis of anticancer studies. All statistical analyses were performed using Prism software (Irvine, CA). A *p* value of <.05 was considered statistically significant.

## 3. Results and discussion

### 3.1. Loading SN-38 in HSA–PLA nanoparticles

The use of HSA, an endogenous protein, as scaffold materials in drug delivery systems has a number of recognized advantages, such as the availability of a variety of functionalized groups, good biocompatibility and biodegradability, and less unexpected side effects during clinical translation (Hong et al. 2020; Chu et al. 2022). The superiority of HSA-based nanoparticles over other nanocarriers has been demonstrated in multiple clinical trials involving paclitaxel formulations. Specifically, alternative nanoplatforms such as polymer–drug conjugates (e.g. XYOTAX), vitamin E-based emulsions (e.g. Tocosol), liposomal paclitaxel (e.g. Lipusu), and polymeric nanoparticles (e.g. NK105) failed to show a significant survival benefit over standard paclitaxel (Xiong, Li, et al. 2025). In contrast, Abraxane, an HSA-based paclitaxel nanoparticle formulation, was approved by the US FDA based on its demonstrated improvement in progression-free survival and low toxicity. These clinical outcomes further support the advantages of using HSA in drug delivery.

Formulating hydrophobic drugs typically requires the use of organic solvents; however, these solvents often denature and precipitate proteins. Therefore, when loading hydrophobic drugs onto HSA-based delivery systems, protein denaturation must be considered. For example, in the synthesis of antibody–SN-38 or HSA–SN-38 conjugates (Moon et al. 2008; Sepehri et al. 2014), SN-38 was modified with a functionalized group and then conjugated to proteins in a hydrophilic medium (typically containing 10% v/v DMSO). We also developed a drug loading method, using a very limited volume of alcoholic solvents to encapsulate hydrophobic drugs into HSA-based drug delivery systems. As described in our previous studies titled ‘Acetyl-lysine human serum albumin nanoparticles activate CD44 receptors, with preferential uptake by cancer stem cells, leading to tumor eradication’ (Xiong, Schätzlein, et al. 2025) and ‘Amphiphilic albumin-based nanoparticles designed for the efficient delivery of taxanes’ (Xiong, Li, et al. 2025), HSA–PLA nanoparticles successfully encapsulated taxanes, including paclitaxel, docetaxel, and cabazitaxel.

**Figure 2.**
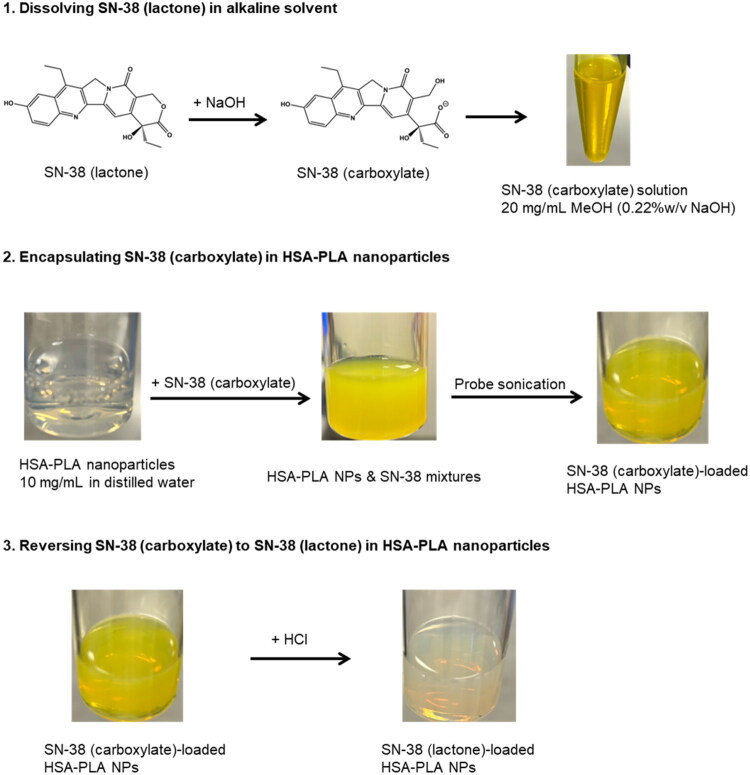
Schematic representation of SN-38 encapsulation in HSA–PLA nanoparticles and lactone–carboxylate conversion. (1) SN-38 (lactone) is dissolved in an alkaline solvent (0.22% w/v NaOH in methanol), transforming into SN-38 (carboxylate). (2) The SN-38 (carboxylate) form is encapsulated into HSA–PLA nanoparticles through probe sonication, resulting in SN-38 (carboxylate)-loaded nanoparticles. (3) The SN-38 (carboxylate) in HSA–PLA nanoparticles is converted back to the lactone form by acidification with HCl, ensuring the active form of SN-38 is retained for drug delivery applications.

In this work, SN-38 was encapsulated in the HSA–PLA nanoparticles with a similar drug loading method. We utilized the fact that the lactone and carboxylate forms of SN-38 may be reversibly interconverted. The ionized groups allow SN-38 carboxylate to be dissolved in more polar solvents, such as MeOH, whereas SN-38 lactone is insoluble. As illustrated in Figure 2, SN-38 carboxylate was dissolved in MeOH (0.22% w/v NaOH) at a concentration of 20 mg/mL, appearing as a bright yellow solution. Subsequently, the SN-38 carboxylate solution was added to the HSA–PLA nanosuspension, resulting in a turbid mixture. After probe sonication, SN-38 carboxylate was encapsulated within the HSA–PLA nanoparticles, yielding a transparent and bright yellow nanosuspension. With the addition of HCl solution, NaOH was neutralized and thus pH was adjusted to neutral (pH = 7). The bright yellow color disappeared, and no drug precipitation was observed, indicating that SN-38 was transformed into its lactone form within the core of the HSA–PLA nanoparticles. Consequently, SN-38 in its lactone form was encapsulated in the HSA–PLA nanoparticles.

Compared to the complicated process of SN-38 modification and conjugation, our method is significantly simpler and highly reproducible. We believe that our drug-loading method offers a promising and clinically translatable approach for formulating SN-38 with protein-based delivery systems.

### 3.2. Characterization of HSA–PLA (SN-38) NPs

**Figure 3.**
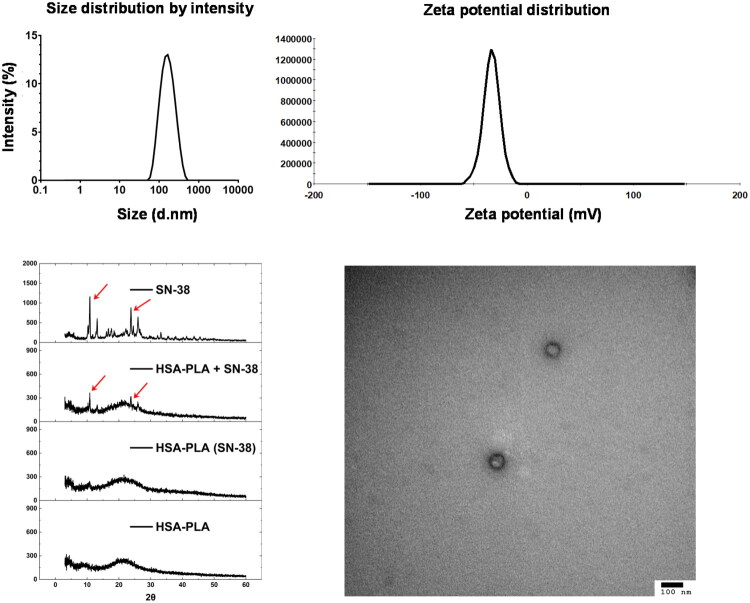
Characterization of SN-38-loaded HSA–PLA nanoparticles. Top left: the size distribution by intensity shows a monodisperse population of nanoparticles with a narrow size range. Top right: zeta potential measurement showing a single negative peak, further confirming the surface uniformity and good colloidal stability of the nanoparticles. Bottom left: X-ray diffraction (XRD) patterns of free SN-38, physical mixture (HSA–PLA + SN-38), SN-38-loaded HSA–PLA nanoparticles and blank HSA–PLA nanoparticles. The disappearance of SN-38 crystalline peaks after encapsulation indicates successful drug incorporation and conversion into an amorphous form. Bottom right: transmission electron microscopy (TEM) image confirms the spherical morphology of the nanoparticles with a uniform size distribution (scale bar: 100 nm).

The hydrodynamic diameters and zeta-potentials of lyophilized HSA–PLA (SN-38) nanoparticles were analyzed using DLS. As listed in Table 1 and shown in the size distribution graph (Figure 3), the particle size was 163 ± 21 nm, with a PDI of 0.18 ± 0.04, indicating a uniform size distribution. The zeta potential of the nanoparticles was −32.4 ± 1.4 mV, suggesting good colloidal stability in physiological medium. Transmission electron microscopy images further confirmed the spherical morphology of the nanoparticles, as illustrated in Figure 3.

**Table 1.** Characterization parameters of SN-38 loaded HSA–PLA nanoparticles.

Parameters	HSA–PLA (SN-38)
Hydrodynamic diameters (nm)	163 ± 21
PDI	0.18 ± 0.04
Zeta-potentials (mV)	−32.4 ± 1.4
DL% w/w	19.4 ± 0.5
EE% w/w	97.1 ± 2.6

Data are presented as mean ± SD (*n* = 3).

XRD analysis (Figure 3) revealed several characteristic peaks of SN-38 crystals in both SN-38 standard and physical mixture of SN-38 with HSA–PLA nanoparticles. These peaks were absent in the XRD pattern of the SN-38-loaded nanoparticles, indicating that no SN-38 crystals remained after encapsulation and lyophilization. The LC% and EE% of SN-38 in HSA–PLA nanoparticles were determined using HPLC to be 19.4 ± 0.5% and 97.1 ± 2.6%, respectively.

These physicochemical characterizations collectively confirm the successful encapsulation of SN-38 within the HSA–PLA nanoparticles. By exploiting the lactone–carboxylate equilibrium of SN-38, we have developed a SN-38 loading strategy that markedly reduces formulation complexity compared to conventional SN-38 conjugation approaches. The high EE% and LC% results further support the potential of this method as a simple, cost-effective, and scalable platform for SN-38 delivery, with strong prospects for clinical translation.

### 3.3. Cellular uptake – macropinocytosis pathway

Albumin-based nanoparticles have been extensively studied since the approval of Abraxane in 2005. Their endocytic pathways have also been widely explored, with macropinocytosis identified as one of the primary routes of uptake and lysosomal degradation in cancer cells (Liu and Ghosh 2019). One of the endocytic pathways for HSA–PLA nanoparticles in human cancer cells has been confirmed to be macropinocytosis in previous work (Xiong, Schätzlein, et al. 2025).

**Figure 4.**
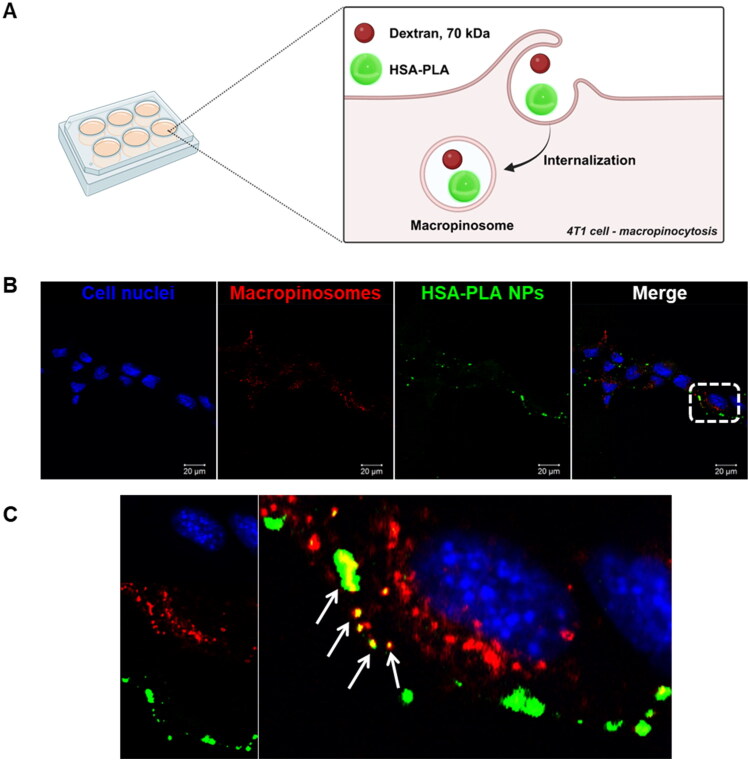
Internalization of HSA–PLA nanoparticles via macropinocytosis in 4T1 cells. Schematic (A) illustrates the uptake mechanism of HSA–PLA nanoparticles (green) into 4T1 cells through macropinocytosis, where they become entrapped within macropinosomes (labeled with dextran). Confocal microscopy images (B) show 4T1 cells incubated with FITC-labeled HSA–PLA nanoparticles (green) and Texas Red–dextran (red), a marker for macropinosomes. Cell nuclei were stained with Hoechst 33342 (blue). Scale bars = 20 µm. Panel (C) displays a zoomed-in confocal image of the white-boxed region. Co-localization of HSA–PLA nanoparticles with macropinosomes is indicated by yellow signals in the merged image (white arrows), confirming macropinocytosis as a major pathway for nanoparticle uptake.

In this study, we further confirmed that macropinocytosis is a primary pathway for the uptake of HSA–PLA nanoparticles in the murine cancer cell line 4T1. As illustrated in Figure 4(A), Texas Red–dextran, a known marker for macropinosomes (Liu and Ghosh 2019), was used to label macropinocytic vesicles. The observed co-localization of HSA–PLA (FITC) nanoparticles with Texas Red–dextran in 4T1 cells confirms that macropinocytosis is a major pathway for the uptake of HSA–PLA nanoparticles in these cells (Figure 4(B,C)).

Cancer macropinocytosis is a nonspecific endocytic pathway in which cells internalize extracellular nutrients to support proliferation, via F-actin-driven membrane ruffling and the formation of large vesicles known as macropinosomes (Puccini et al. 2022). Notably, macropinocytosis pathway is upregulated in various cancers, such as gastrointestinal cancers, breast cancer, and pancreatic cancer, through multiply oncogenic mutations (Puccini et al. 2022). This universal and nonspecific uptake pathway in cancer cells is particularly advantageous for drug delivery. By exploiting macropinocytosis, HSA–PLA nanoparticles can effectively deliver their cargo to a variety of cancer types, demonstrating broad spectrum in cancer drug delivery.

### 3.4. Cell death pathway – apoptosis

SN-38 is a clinically approved chemotherapeutic agent known as a Top I inhibitor. Its mechanism of action has been extensively studied in various cancer cell types, where it stabilizes the Top I–DNA complex, thereby arresting cell cycle and promoting apoptosis (Poele and Joel 1999; Ueno et al. 2002).

**Figure 5.**
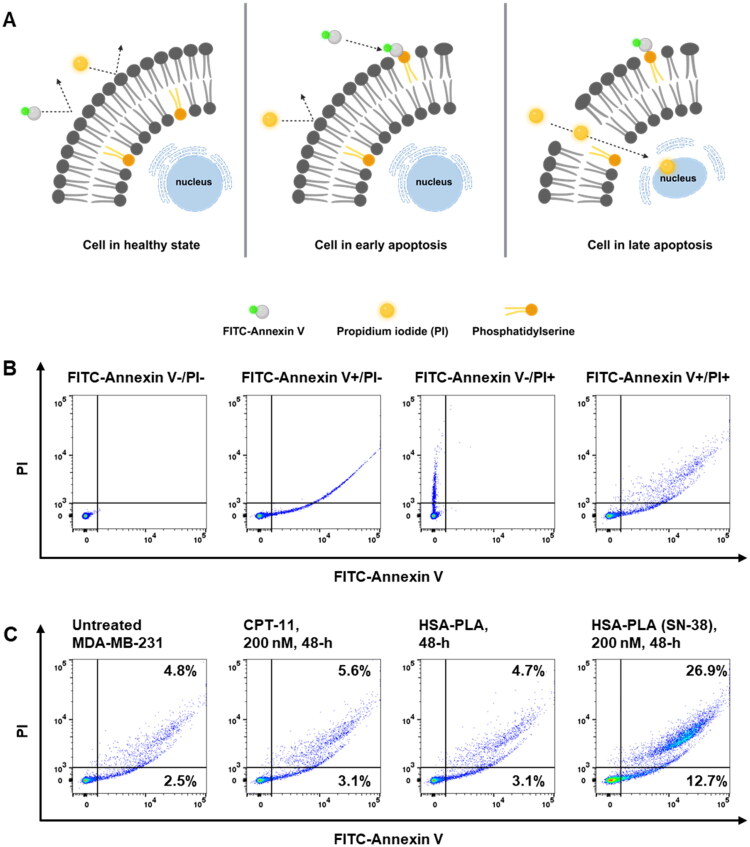
Apoptosis analysis of MDA-MB-231 cells after treatment with CPT-11 and HSA–PLA (SN-38) nanoparticles. (A) Schematic illustration of apoptosis detection using FITC-annexin V and propidium iodide (PI). In healthy cells (left), the phosphatidylserine (PS) remains on the inner leaflet of the plasma membrane. In early apoptotic cells (middle), PS is translocated to the outer membrane, allowing annexin V binding, while the membrane remains intact and impermeable to PI. In late apoptotic or necrotic cells (right), both annexin V and PI can bind due to compromised membrane integrity. (B) Method validation for apoptosis detection using flow cytometry. The first dot plot (from left to right) shows the unstained control (Annexin V−/PI−). The second plot represents cells stained with Annexin V-FITC only. The third plot shows cells stained with PI only. The final plot displays the distribution of cells stained with both dyes. (C) Dot plots (from left to right) represent the untreated cell control, cells treated with 200 nM CPT-11 for 48 hours, cells treated with the vehicle (HSA–PLA), and cells treated with HSA–PLA (SN-38) at 200 nM for 48 hours. Cells treated with HSA–PLA (SN-38) showed a significant increase in the proportion of apoptotic cells.

It has been reported that nanoformulating chemotherapeutic agents may alter the mechanisms of programmed cell death in cancer cells (Luobin et al. 2024). In this work, the Annexin V and PI apoptosis assay was applied to validate if the SN-38 loaded HSA–PLA nanoparticles could induce cell apoptosis. As illustrated in Figure 5(A), Annexin V and PI cannot bind to healthy cells. In early apoptosis, Annexin V binds to phosphatidylserine (PS) exposed on the outer leaflet of the cell membrane. PI is a DNA-binding dye that can only penetrate cells with compromised plasma membranes and thus stains cells in late apoptosis (Kari et al. 2022). We have validated the suitability of this apoptosis assay by comparing flow cytometry dot plots of MDA-MB-231 cells in the absence or presence of FITC-Annexin V and PI. As illustrated in Figure 5(B), when MDA-MB-231 cells were not stained with FITC-Annexin V and PI, no events were detected in either the FITC or PI channels. After staining cells with FITC-Annexin V only, apoptotic cells were detected in the FITC channel, along with some undesired signals in the PI channel. When cells were stained with PI alone, late apoptotic cells were detected in the PI channel. After staining with both FITC-Annexin V and PI, signals corresponding to both early and late apoptotic cells were observed in the right panel. Although part of FITC emission was counted into the PI channel, this interference did not affect the calculation of total apoptotic cells, as distinguishing between early and late apoptosis was not required in this study.

After treating MDA-MB-231 cells with 200 nM CPT-11 or HSA–PLA (SN-38) for 48 hours, the proportion of apoptotic cells was assessed using FITC-Annexin V and PI staining. Untreated cells and cells treated with blank HSA–PLA nanoparticles served as control groups. The proportion of apoptotic cells in the untreated group was 8.0 ± 1.5%, which was not statistically different from that in cells treated with CPT-11 (9.9 ± 1.0%) or blank HSA–PLA nanoparticles (8.9 ± 2.3%). Cells treated with HSA–PLA (SN-38) have shown a significant increase in apoptotic cells (34.9 ± 4.1%) when compared to the untreated, CPT-11 and HSA–PLA groups, *p* < .0001.

This apoptosis study confirmed that SN-38-loaded HSA–PLA nanoparticles can effectively induce cell apoptosis at low concentrations. By using the nonspecific cellular uptake pathway of HSA–PLA nanoparticles, namely macropinocytosis, this delivery system may enable SN-38 to achieve broader chemotherapeutic applicability compared to CPT-11 in colorectal cancer.

### 3.5. *In vitro* cytotoxicity

It is known that CPTs, in their carboxylate forms, lose their biological activity, resulting in reduced efficacy in cancer therapy (Khaiwa et al. 2021). Therefore, formulating CPTs presents the challenge that CPT lactones can be rapidly hydrolyzed into an inactive form, CPT carboxylates, under physiological conditions (37 °C, pH >7, and in the presence of albumin or serum proteins) (Burke and Mi 1993). Although the addition of 7-ethyl and 10-hydroxy groups could reduce the conversion from lactone to carboxylate compared to CPT, more than 60% of SN-38 (7-ethyl-10-hydroxycamptothecin) was still hydrolyzed (Burke and Mi 1993).

**Figure 6.**
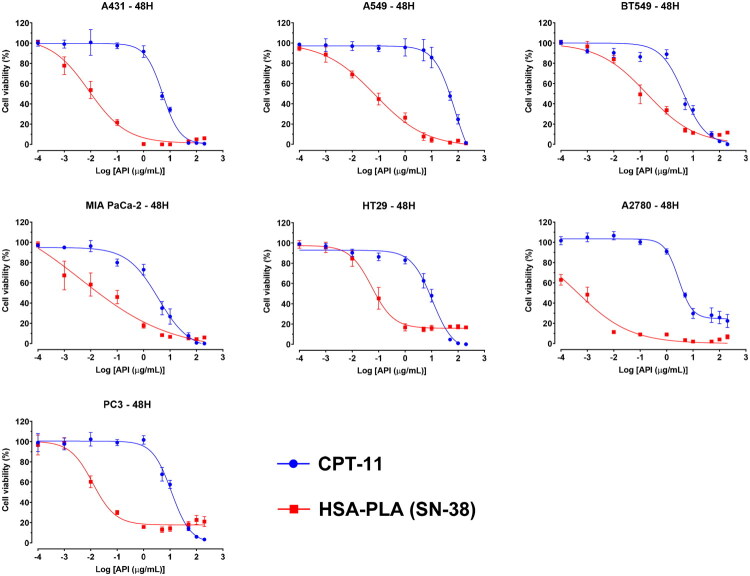
Cytotoxicity of CPT-11 and HSA–PLA (SN-38) nanoparticles in various cancer cell lines. Dose–response curves show the cytotoxic effects of CPT-11 (blue) and HSA–PLA (SN-38) nanoparticles (red) on different cancer cell lines (A431, A549, BT549, MIA PaCa-2, HT29, A2780, and PC3) after 48 hours of treatment. Cell viability (%) was assessed using a WST-1 assay, and drug concentrations are expressed as the logarithm of active pharmaceutical ingredient (API) concentrations (µg/mL). HSA–PLA (SN-38) nanoparticles exhibit a greater cytotoxic effect compared to CPT-11 across all tested cell lines, indicating enhanced potency. Error bars represent standard deviations from triplicate experiments.

Therefore, we treated cancer cells with HSA–PLA (SN-38) nanoparticles in complete medium (in the presence of FBS) for 48 hours to evaluate the cytotoxicity of this SN-38 formulation under physiological conditions. As shown in the dose–response curves in Figure 6, HSA–PLA (SN-38) nanoparticles exhibited an enhanced cytotoxic effect compared to CPT-11 across various cancer cell lines, including A549, PC3, BT549, HT29, A2780, A431, and MIA PaCa-2. The IC_50_ values for SN-38-loaded HSA–PLA nanoparticles range from 0.0005 µg/mL to 0.194 µg/mL (0.001–0.494 µM), which are significantly lower than those of CPT-11, ranging from 2.5 µg/mL to 104 µg/mL (6.37–265.04 µM). Details are listed in Table 2.

**Table 2.** IC_50_ (µg/mL) of CPT-11 and HSA–PLA (SN-38) in various cancer cell lines.

Cell line	CPT-11	HSA–PLA (SN-38)	*p* Value
A2780	2.8 ± 0.3	0.0005 ± 0.0002	<.0001
A431	5.3 ± 0.1	0.010 ± 0.008	<.0001
A549	104.7 ± 63.8	0.074 ± 0.021	.047
BT549	4.4 ± 1.7	0.194 ± 0.107	.013
HT29	10.8 ± 1.7	0.060 ± 0.029	.0004
MIA PaCa-2	2.5 ± 0.9	0.035 ± 0.029	.009
PC3	11.5 ± 1.8	0.012 ± 0.001	.0004

Data are presented as mean ± SD (*n* = 3) and analyzed with multiply *t*-test. A *p* value <.05 is considered significantly different.

These findings demonstrate the remarkable potency of the HSA–PLA (SN-38) nanoparticles in a broad spectrum of cancer cell lines, significantly outperforming the clinical standard CPT-11. The consistently low IC_50_ values suggest that the encapsulated SN-38 remains biologically active even in serum-containing conditions, where lactone ring hydrolysis typically compromises the efficacy of CPT-based drugs. This implies that our encapsulation formulation may help preserve the lactone form of SN-38, at least in part, by shielding it from premature hydrolysis or binding to serum albumin.

The superior *in vitro* anticancer potency of HSA–PLA (SN-38) nanoparticles across various cancer cell lines may also be attributed to their efficient, nonspecific uptake via macropinocytosis and their ability to induce apoptosis at low concentrations. Coupled with this broad cytotoxic activity, formulating SN-38 with our strategy has shown a promising potential for broadening its application in cancer therapy, especially as the clinical indication of CPT-11 is primarily for colorectal cancer.

### 3.6. *In vivo* anticancer studies

The *in vivo* efficacy of HSA–PLA (SN-38) nanoparticles was evaluated in a murine breast cancer model (4T1 tumors in immunocompetent mice) and humanized breast cancer model (MDA-MB-231 tumors in immunocompromised mice).

**Figure 7.**
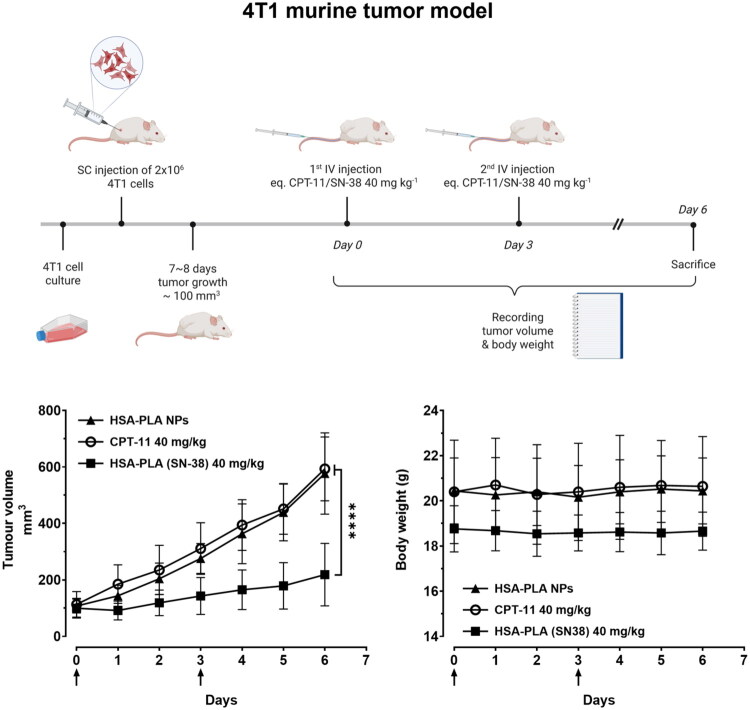
*In vivo* antitumor study in 4T1 tumor bearing models. The top illustration provides a brief overview of the *in vivo* study design. Created with biorennder.com. The bottom left panel shows tumor volume growth over time in mice treated with HSA–PLA nanoparticles (control), CPT-11 (40 mg/kg), and HSA–PLA (SN-38) nanoparticles (40 mg/kg). The HSA–PLA (SN-38) nanoparticles exhibited a significantly greater tumor suppression effect compared to CPT-11 and the control group (****p < .0001). Data are shown as mean ± standard deviation (SD), *n* = 5. The bottom right panel presents body weight measurements over the study period, demonstrating no significant weight loss in any treatment group, indicating the safety of the formulations. Arrows indicate the time of drug administration. Data are presented as mean ± SD, *n* = 5.

4T1 tumor-bearing mice were treated with either CPT-11 or HSA–PLA (SN-38) nanoparticles (both at a dose of 40 mg/kg) via two intravenous injections. As shown in Figure 7, the HSA–PLA (SN-38) nanoparticle-treated group (*n* = 5) exhibited significantly greater tumor growth inhibition compared to blank nanoparticles and the CPT-11-treated group (*n* = 5), *p* < .0001. The stable body weight records of HSA–PLA (SN-38) treated mice are indicative that it might be safe for use at a dose of 40 mg/kg in mice. No signs of hypersensitivity or weight loss were observed in control mice receiving two injections of HSA–PLA blank nanoparticles.

**Figure 8.**
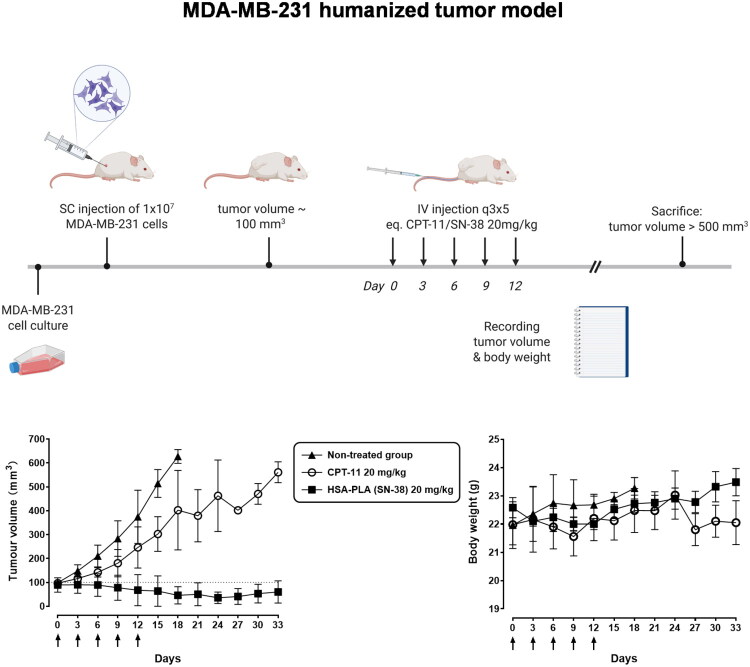
Tumor growth inhibition and body weight monitoring in the MDA-MB-231 humanized tumor model. (Top) A schematic overview of the anticancer study conducted in the humanized tumor model (MDA-MB-231). Created with biorennder.com. (Bottom left) Tumor volume progression over 33 days in mice treated with HSA–PLA (SN-38) nanoparticles (20 mg/kg), CPT-11 (20 mg/kg), or left untreated. The HSA–PLA (SN-38) group exhibited a significant reduction in tumor growth compared to the non-treated and CPT-11-treated groups. Data are presented as mean ± SD, *n* = 5. (Bottom right) Body weight measurements over the same period, showing no significant weight loss across all groups, indicating good tolerability of the treatments. Arrows indicate the time points of drug administration. Data are presented as mean ± SD, *n* = 5.

As depicted in Figure 8, in MDA-MB-231 tumor-bearing mice, the non-treated control group exhibited a rapid increase in tumor volume, reaching approximately 600–700 mm^3^ by day 18. After five doses of CPT-11 (20 mg/kg), MDA-MB-231 tumor growth was slightly suppressed. The fluctuation in the tumor growth curve for the CPT-11 treatment group was due to the removal of mice when their tumor volumes exceeded the humane endpoints. In contrast, mice treated with HSA–PLA (SN-38) nanoparticles (*n* = 5) demonstrated significant tumor growth suppression compared to both the control and CPT-11-treated groups. Tumor volumes in the HSA–PLA (SN-38) group remained stable and significantly lower on day 33, suggesting superior efficacy of this formulation. Meanwhile, the HSA–PLA (SN-38) group maintained stable body weights throughout the study, indicating good tolerability at the administered multiple doses of 20 mg/kg.

The dosage selection was carefully tailored to each tumor model based on the immune background of the host mice and animal welfare considerations. In the 4T1 model, tumors were implanted in immunocompetent BALB/c mice, which are capable of mounting immune responses against xenogeneic proteins. As HSA is a human-derived protein, repeated intravenous administration can potentially trigger hypersensitivity reactions in this model. To minimize the risk of immunogenicity and ensure animal welfare, we employed a reduced injection frequency with a higher single dose (40 mg/kg SN-38 equivalent). In contrast, the MDA-MB-231 model was established in NOD-SCID mice, which lack functional T and B lymphocytes and therefore do not mount an adaptive immune response against human proteins. This allowed us to adopt a multiple low-dose administration strategy (20 mg/kg, more frequent injections) to better assess the therapeutic effect over time.

SN-38-loaded HSA–PLA nanoparticles significantly inhibited both murine and humanized tumor growth in mice compared to the existing drug, CPT-11, demonstrating strong translational potential. Our previous work has confirmed that HSA–PLA based nanoparticles can effectively and safely deliver therapeutic cargoes to tumors (Xiong, Li, et al. 2025; Xiong, Schätzlein, et al. 2025). Detailed toxicology evaluations and additional antitumor studies on HSA–PLA (SN-38) nanoparticles will be conducted in future work.

## 4. Conclusions

SN-38 is an important clinical agent for cancer therapy. However, a suitable delivery approach for SN-38 using a HSA platform is not yet available for clinical use, as SN-38 lactone is hard to formulate.

In this work, we found that SN-38 carboxylate may be dissolved in alcohols and easily encapsulated in the HSA-based nanoparticles. By exploiting the SN-38 lactone–carboxylate equilibrium, we reconverted the SN-38 carboxylate to the SN-38 lactone in the cores of HSA–PLA nanoparticles, achieving a high drug loading of 19% w/w. This encapsulation strategy effectively maintained the cytotoxic activity of SN-38 at low concentrations under physiological conditions. The macropinocytosis-mediated, nonspecific uptake of HSA–PLA nanoparticles by various cancer cells ensures the broad-spectrum applicability of SN-38 when encapsulated within these nanoparticles. The superior *in vitro* and *in vivo* efficacy of SN-38-loaded HSA–PLA nanoparticles over CPT-11 further demonstrate that this drug-loading strategy effectively addresses the challenges associated with proper SN-38 formulation.

Overall, this work presents an alternative and promising strategy for the effective delivery of SN-38 using HSA–PLA nanoparticles and supports its potential for clinical translation.

## Data Availability

Data will be made available on request from the corresponding author.
